# Study on Vertical Bearing Capacity of Pile Foundation with Distributed Geopolymer Post-Grouting on Pile Side

**DOI:** 10.3390/ma17020398

**Published:** 2024-01-12

**Authors:** Pan Li, Yangyang Xia, Xinhui Xie, Jing Wang, Chaojie Wang, Mingsheng Shi, Bo Wang, Haoye Wu

**Affiliations:** 1 Yellow River Laboratory, School of Water Conservancy and Transportation, Underground Engineering Research Institute, Zhengzhou University, Zhengzhou 450001, China; lipan_bridge@126.com (P.L.); wang925jing@126.com (J.W.); wangyichaojie123@163.com (C.W.); sms315@126.com (M.S.); 2 Henan Yellow River Expressway Co., Ltd., Zhengzhou 450000, China; 3 Yellow River Institute of Hydraulic Research, Research Center for Embankment Safety and Disaster Prevention Engineering Technology of Ministry of Water Resources, Zhengzhou 450003, China; 4 POWERCHINA Guiyang Engineering Co., Ltd., Guiyang 550081, China; wangb_gyy@powerchina.cn; 5 College of Letters and Science, University of California, Davis, CA 95616, USA; wuhaoye747502@163.com

**Keywords:** geopolymer, pile side-distributed grouting piles, model test, bearing capacity, diffusion form

## Abstract

To study the applicability of the new geopolymer grouting material for super-long and large-diameter post-grouting bored piles in silty fine sand geology, this paper compares the bearing capacity of two grouting materials, geopolymer and normal Portland cement, and different grouting volume pile side-distributed grouting piles in silty fine sand based on field model tests are analyzed through the diffusion forms of the two materials in silty fine sand through the morphology of the grouted body after excavation. The results show that the ultimate bearing capacities of P0 (ungrouted pile), P1 (8 kg cement grouted pile), P2 (6 kg geopolymer-grouted pile), P3 (8 kg geopolymer-grouted pile) and P4 (10 kg geopolymer-grouted pile) are 5400 N, 8820 N, 9450 N, 11,700 N and 12,600 N, respectively, and that the ultimate bearing capacity of the grouted pile is improved compared with that of the ungrouted pile since, under the same grouting amount, the maximum bearing capacity of the pile using geopolymer grouting is increased by 133% compared with that of the pile with cement grouting. This further verifies the applicability of the geopolymer grouting material for the post-grouting of the pile foundation in silty fine sand. Under the action of the ultimate load, the pile side friction resistance of P1, P2, P3 and P4 is increased by 200%, 218%, 284% and 319% compared with that of P0. In addition, the excavation results show that the geopolymer post-grouting pile forms the ellipsoidal consolidation body at the pile side grouting location, which mainly comprises extrusion diffusion with a small amount of infiltration diffusion, and the cement grouting pile forms a sheet-like consolidation body at the lower grouting location, which primarily comprises split diffusion. This study can provide a reference basis for the theoretical and engineering application of post-grouting piles using geopolymers.

## 1. Introduction

**Figure 1:**
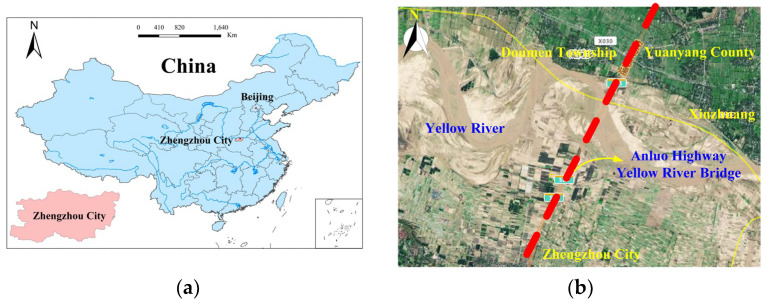
Project location and surrounding environment of (**a**) project location and (**b**) project surrounding environment.

This study relies on the Yellow River super bridge project (Figure 1) of the Yuanyang to Zhengzhou section of the AnLuo expressway that is located in the Yellow River alluvial plain with perennial yellow sand siltation and a super-thick sandy soil layer covering the riverbed, which is prone to liquefaction under complex loads. In order to meet the bearing capacity requirements and control the upper settlement, the project adopts super-long- and large-diameter post-grouting bored piles for the bridge abutment’s foundation [1,2]. Due to the inherent defects of bored piles, such as mud skin on the pile side, slag at the pile end and stress relaxation in the soil around the pile, their bearing capacity is significantly reduced; to solve such problems, technology for the post-grouting of the pile foundation is widely used due to its good engineering benefits [3,4,5].

Super-long and large-diameter post-grouting bored piles exhibit typical friction pile characteristics. Some studies have shown [6] that the end resistance of super-long piles under maximum load without grouting and post-grouting piles only accounts for 5.0% and 10.5% of the top load, respectively, and the effect of post-grouting on end resistance is not apparent. Pile-side grouting can meet the bearing capacity demand of super-long piles.

Pile-side post-grouting technology has been developed over the years; different grouting processes have been developed, e.g., Mullins et al. [7] developed a pile-side grouting device with a vertical porous tube wrapped in rubber film. Sze et al. [8] introduced a device with a grouting flower tube welded on the outside of the reinforcement cage. Nguyen et al. [9] introduced a pile-side grouting device that is composed of a grouting core tube and two upper and lower sealing airbags. During grouting, the device is lowered to the grouting section of the grouting outer tube, inflated to seal and then the slurry is injected into the soil around the pile. Studies have shown that the bearing capacity of grouting piles is twice that of non-grouting piles in sand. In these studies, the researchers temporarily blocked the outlet hole with a rubber film before using a pressure grouting device. Thiyyakkandi et al. [10] designed a pile-side grouting device consisting of multiple grouting pipes of different lengths that can be designed according to different grouting areas. The outlet holes are left at a certain distance in the lower part of each grouting pipe and wrapped with elastic rubber. Huang et al. [11] introduced a pile-side grouting device, which consisted of a vertical steel pipe, to convey the slurry into a horizontal pipe. Zhang et al. [12] introduced an annular pipe-type grouting device that has multiple annular pipes in the pile body. A vertical steel pipe connected each annular pipe. The slurry entered the annular pipe from the vertical pipe, and there was a one-way grouting head distributed on the annular pipe. Fiscina et al. [13] developed a new micro-steel pipe pile-side grouting device in which the small-diameter steel pipe on the pile side directly punched holes to be used as grouting holes. After the steel pipe pile was driven into the ground, the grouting pipe was placed at the location of the steel pipe pile grouting holes and blocked from above so that the slurry could be injected in sections, which made the slurry distributed more uniformly. With the continuous improvement in the post-pile grouting process, the most commonly used methods are the straight pipe method, with a grouting straight pipe placed along the longitudinal direction of the reinforcement cage, and annular pipe grouting, with a grouting annular pipe placed along the inner wall of the reinforcement cage [14]. However, the traditional annular tube-type grouting has problems, such as sizeable grouting spacing, uneven slurry distribution, and the inability to undertake directional grouting [15], which limit the application of post-grouting on the side of super-long piles. Based on this, Dai et al. [15] developed a pile-side distributed post-grouting device, which has the advantages of a flexible grouting section, uniform slurry distribution and controllable grouting pressure, and the same grouting pipe can be pressed with different slurries than the traditional pile-side grouting and has good applicability to the side post-grouting of super-long bored piles.

Due to the defects of in situ field tests, such as being time-consuming, costly and providing subsurface conditions, the test conditions are limited. The indoor model tests make up for the shortcomings of field tests well and offer a more convenient approach to systematically study the bearing mechanism of post-grouting piles. Their advantages include short time consumption, low cost, controllable test conditions and better reflection of the real traits. Based on the similarity theory, Zhou et al. [16] carried out a centrifugal model test of post-grouting pile group in the loess area. By changing the pile spacing and the number of piles, the bearing capacity of the pile group before and after grouting was analyzed, and we proposed a calculation method for the bearing capacity of post-grouting pile groups suitable for the loess areas. Zhao et al. [17] analyzed the effect of different volumes of the pile end. Baca M. et al. [18,19,20] carried out the indoor model test of the bearing capacity of pipe piles in sandy soil, and they introduced a new bi-directional static load testing method for pipe piles. In this test, the capacity of the pile base and shaft can be measured separately without the necessity of building a retaining structure. The differences between different test methods are compared and analyzed, and then the finite element numerical models of pipe piles with different sizes are established. The applicability of the method is verified by comparing it with the field test results. Wan et al. [21] used aluminum tubes as model piles to compare the vertical bearing mechanism of ungrouted piles, ring-point grouted piles and directional grouted piles in calcareous sand, and the results showed that grouting on the pile side could solidify the soil around the pile and improve the pile side frictional resistance, and the vertical bearing performance of directional grouted piles was better than that of ring-point grouted piles. Zhang et al. [22] used a self-made model pile to simulate the cast-in-place bored pile and studied the influence of different grouting methods and different grouting parameters on the bearing capacity of the model pile in clay. The results show that the bearing capacity of the combined grouting pile is the best, pile-side grouting can greatly reduce the settlement of the pile top and the grouting pressure has little effect on the bearing capacity. Wu et al. [23] investigated the bearing mechanism of post-grouting concrete model piles in silty soils by considering the unloading effect of the soil during pile formation, and they found that post-grouting at the pile end can effectively improve the adverse effects caused by soil unloading, and the height of slurry upward return increases with the increase in the unloading degree. The results show that the pile-side jet grouting improves the horizontal bearing capacity of the pile most, followed by the pile-side distributed grouting, and the ring point grouting is the smallest.

At present, most of the post-grouting materials for bored piles are mainly cement slurry. However, traditional cement slurry has several disadvantages, including slow setting and hardening, significant drying shrinkage, weak bonding performance and high production energy consumption [24]. These drawbacks limit the application of cement slurry in post-grouting. The Yellow River Super Bridge passes through the Yellow River Wetland Reserve. The geological conditions are complex, and the environmental protection requirements are high. Traditional cement slurry cannot meet the high-quality development requirements of the Yellow River Basin. Therefore, there is a need to identify a new grouting material to replace the traditional cement slurry. As a new type of green material, geopolymers have excellent mechanical properties, durability and environmental friendliness. Compared with normal Portland cement, the mechanical properties of geopolymers not only meet the specification requirements but also have high early strength, low permeability, good acid and alkali resistance, excellent bonding properties and good fluidity [25,26]. In addition, the constituents of geopolymers, such as fly ash and blast furnace slag, are derived from industrial waste. The synthesis of geopolymers not only facilitates efficient waste utilization but also contributes to an 80% reduction in carbon emissions compared to conventional cement production [27,28,29]. This presents substantial economic value and environmental advantages for the sustainable development of the Yellow River Basin.

Geopolymers have found extensive applications in the field of construction engineering. For instance, Silva et al. [30] conducted a study in a chloride ion environment, revealing that geopolymer grouting materials effectively shield reinforcement from corrosion and exhibit robust resistance to acid and alkali corrosion. Zhang et al. [31] proved that geopolymer concrete has better interfacial bonding properties through interfacial strength tests on plain concrete and geopolymer concrete bonded to reinforcement. Rios et al. [32] delved into the mechanical properties and microstructural characteristics of fly ash-based geopolymer-cured chalk, and the results showed that fly ash-based geopolymer can effectively improve the unconfined compressive strength and structural denseness of chalk, and the shear damage form of fly ash-based geopolymer-cured chalk soil was found to be similar to that of cement-cured chalk soil. Xiong et al. [33] pioneered the development of a geopolymer grouting material for reinforcing surrounding rock in basements and roadways. The results show that the geopolymer grouting slurry can penetrate the surrounding rock cracks and fully cement with the surrounding rock, which can effectively reduce the deformation of the surrounding rock and improve the bearing capacity of the surrounding rock. Guo et al. [34] devised a cost-effective geopolymer grouting slurry for repairing key formation cracks in aquifers. The repaired formation has higher strength and impermeability, and the repair effect is much higher than that of ordinary cement grouting slurry. In summary, geopolymers enjoy widespread use in engineering applications, serving as grouting materials, cementitious materials and repair materials [35,36]. However, there are few reports on the application of geopolymers as grouting materials in the field of the post-grouting of pile foundations.

In conclusion, considering the limitations in the existing research on the vertical bearing capacity of geopolymer post-grouting piles, this paper conducted a model test to evaluate the vertical bearing capacity of distributed post-grouting super-long bored piles along the pile side. The vertical bearing characteristics of super-long bored piles in silty fine sand were examined under varying grouting materials and amounts. A comparative analysis of the diffusion patterns among different groups in the sand was conducted based on the excavation results of post-grouting model piles. This investigation serves as a valuable reference for advancing the theory and practical application of geopolymer post-grouting piles.

## 2. Model Tests

### 2.1. Model Test Tank

**Figure 2:**
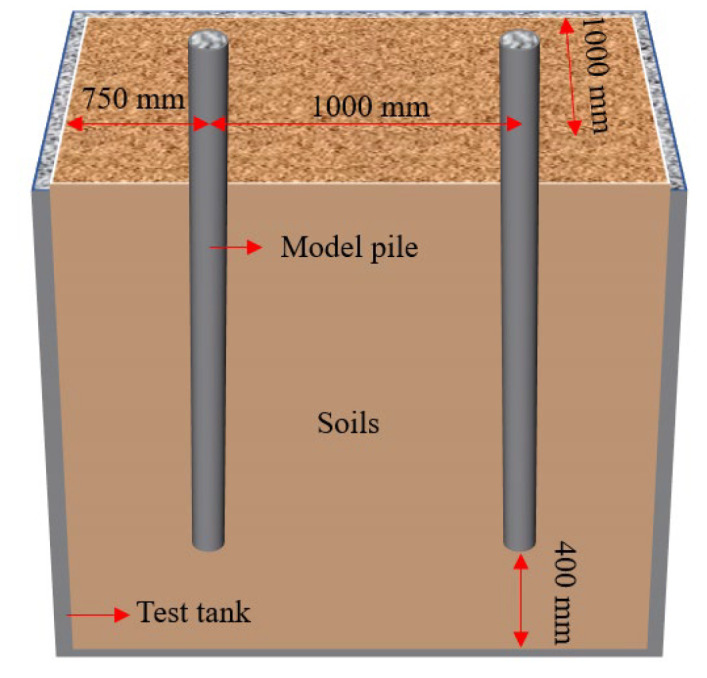
Model pile layout diagram.

The utilization of model testing proves to be an effective approach for investigating the bearing capacity of post-grouting piles [37]. In this study, a purpose-designed concrete test tank, illustrated in Figure 2, was crafted. The wall thickness of the test tank is 30 cm, and the size of the inner wall is 2 m × 2.5 m × 2.5 m (length × width × depth). Drainage holes are strategically positioned at the bottom of the side wall of the test tank to facilitate proper drainage. The potential influence of boundary effects between the model pile and the inner wall of the test groove cannot be overlooked in the arrangement of the model pile. Referring to pertinent literature [38], the design of the model box and the model pile adheres to the necessary test requirements. As per the experimental setup, two model piles are incorporated into each test. The distance between the two model piles is 1 m. The minimum distance between the model pile and the inner wall of the test tank measures 72 cm, constituting 13.3-times the model’s size and satisfying the stipulated boundary requirements. The layout of the model pile is shown in Figure 1.

### 2.2. Model Pile Preparation

**Figure 3:**
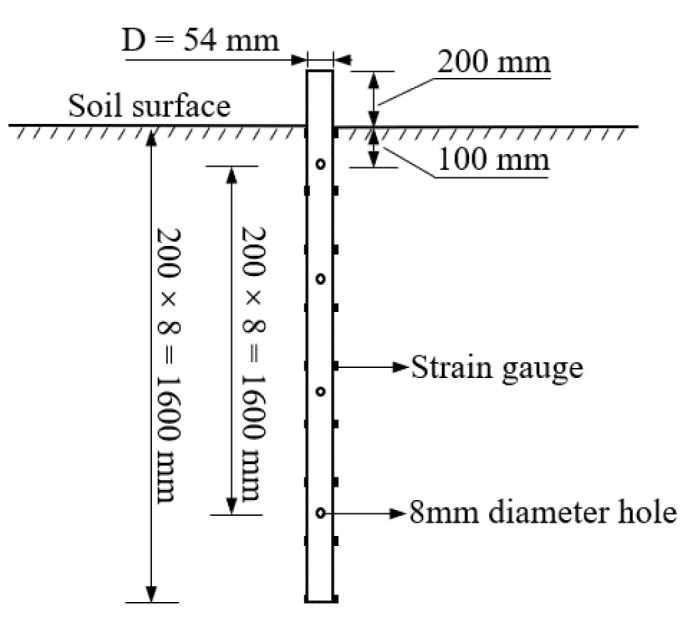
Model pile schematic diagram.

In order to simulate the super-long and large-diameter cast-in-place piles of the Yellow River Large Bridge, piles with a length of 80 m and a diameter of 2.7 m are taken as the research object. This test is based on the similarity principle and the geometric similarity constant C = L_s_/L_m_ = 50. L_s_ and L_m_ are the sizes of the field engineering and model piles, respectively. Consequently, a galvanized hollow steel pipe with a pile length of 1800 mm, an outer diameter of 54 mm, an inner diameter of 50 mm and a buried depth of 1600 mm was selected as the model pile. The measured elastic modulus of the model pile is E = 205 GPa, with no consideration for the influence of pile surface roughness at this stage. To prevent soil ingress in the model pile, the pile end is sealed with the same material gasket. A schematic diagram of the model pile is presented in Figure 3. Commencing 200 mm from the top of the pile, a pair of resistance strain gauges, model BE120-5AA (11)-P300, is symmetrically arranged along the pile body at intervals of 200 mm downward. In total, nine pairs of strain gauges are strategically placed. Starting from 300 mm from the top of the pile, a pair of grouting holes with a diameter of 8 mm is symmetrically arranged every 400 mm downward, resulting in a total of four grouting sections.

**Figure 4:**
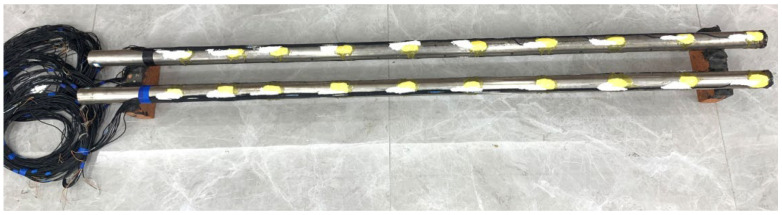
Model pile physical diagram.

To better protect the strain gauge from damage, a layer of elastic waterproof glue is applied to the strain gauge after completion of the pasting process, followed by an outermost coating of epoxy resin (Figure 4). Finally, the strain gauge wire is secured on the pile side using glue, and a layer of insulating waterproof tape is wrapped around the pile side.

### 2.3. Model Soil and Pile Formation Methods

#### 2.3.1. Soil

**Figure 5:**
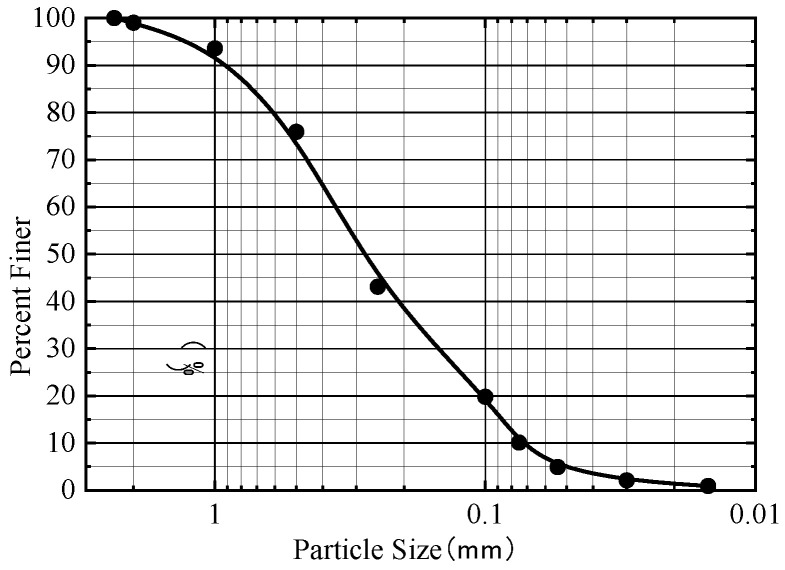
Soil particle gradation curve.

The soil utilized in this test is sourced from the vicinity of the large Yellow River Super Bridge project. Geotechnical tests were conducted in accordance with the industry standard of the People‘s Republic of China (JTG 3430-2020) [39]. The soil parameters obtained from indoor geotechnical tests are presented in Table 1. The particle size distribution curve of the test soil is depicted in Figure 5. It is evident that the percentage composition of sand, silt and clay in the test soil is 88.9%, 10.1% and 0.1%, respectively. The mass of particles with a size greater than 0.075 mm constitutes more than 50% of the total mass, categorizing the test soil as silty fine sand.

#### 2.3.2. Formation of Pile

**Figure 6:**
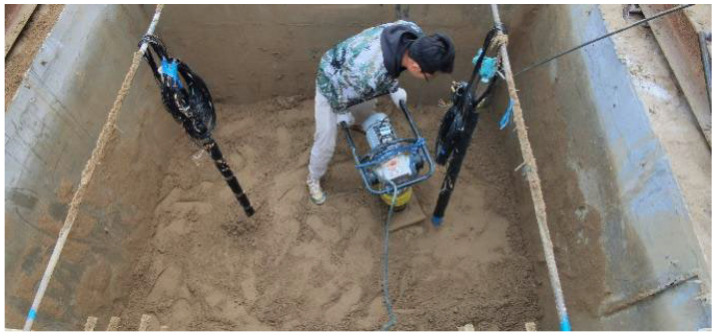
Layered tamping of pre-embedded model piles.

**Figure 7:**
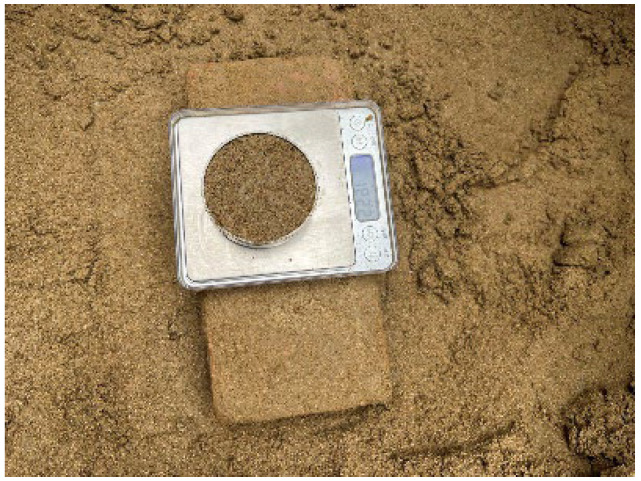
Measuring the density of each fill layer.

This test employs the pre-buried method for pile formation. Initially, fine silt sand is added to the test tank in layers and compacted using a plate-vibrating rammer every 200 mm (Figure 6). When the filling height reaches 400 mm, the lead pendant is utilized to pinpoint the position of the model piles. The slurry holes on the model piles are sealed with adhesive tapes to prevent soil from obstructing them. Subsequently, soil tamping is continued in layers until reaching the designated burial depth of 1600 mm. In the process of burying the pile, we constantly determined the density of each layer of soil (Figure 7) to maintain the soil body density at 1.9 g/cm^3^.

### 2.4. Test Programs and Devices

#### 2.4.1. Grouting Program

**Figure 8:**
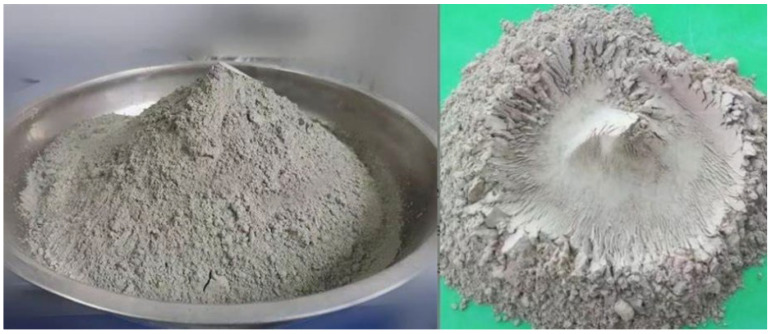
Normal Portland cement (**left**) and CN-I geopolymer (**right**).

**Figure 9:**
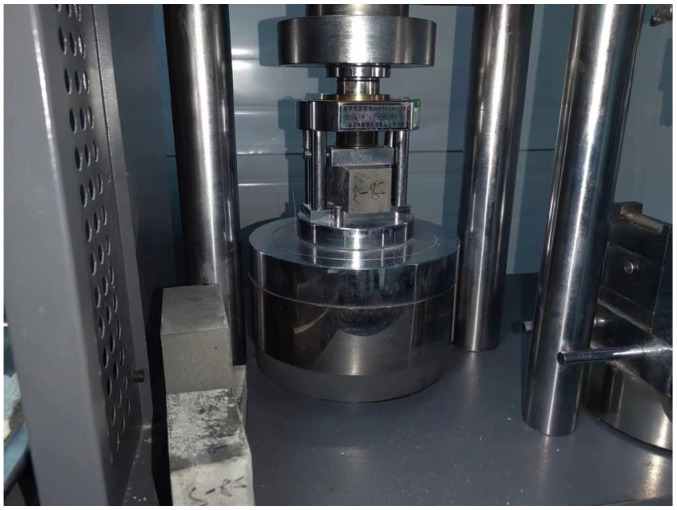
Compressive strength testing.

**Figure 10:**
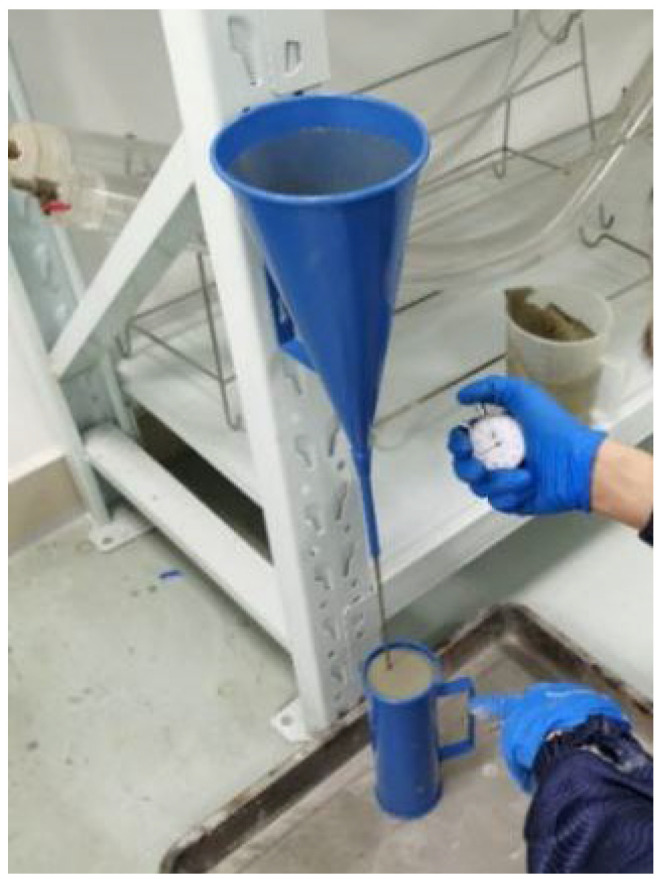
Fluidity test.

This experiment utilized P.O42.5 ordinary Portland cement and CN-I type geopolymer grouting slurry (Figure 8). CN-I type geopolymer grouting slurry was prepared using a specific proportion of metakaolin, fly ash, slag powder and other minerals, activator (powder), early strength agent and expansion agent. It has the characteristics of renewable raw materials, green environmental protection, good fluidity, controllable setting time, high early strength, late strength without shrinkage, micro expansion and good durability. In accordance with the Chinese standard (JTG 3420-2020) [40], the basic mechanical performance parameters are tested, as shown in Table 2, and the compressive strength test is shown in Figure 9. In engineering applications, the flow degree index is an essential factor in judging a grouting material’s injectability. The inverted cone method was used to test the flowability of the two materials (Figure 10), and through several comparisons of the pre-test, it was found that the best grouting effect was achieved when the flowability was 15 s. Therefore, the flowability of the two grouting materials was unified to 15 s. To compare the effects of different grouting quantities on the bearing capacity of model piles, the comprehensive results of the pre-test were utilized. It was determined that the grouting volume was 6 kg, 8 kg and 10 kg in three gradients. The test program is shown in Table 3.

Geopolymer is anticipated to emerge as a prominent substitute for cement in the 21st century, given its comparable mechanical properties and distinctive advantages. The CN-1-type geopolymer slurry employed in this study exhibits several advantages over ordinary cement:

1. It has better injectability. Under a uniform flow degree (15 s), the water secretion rate of geopolymer slurry is less than 0.4%, while the water secretion rate of ordinary cement is more than 2%. The higher water secretion causes the cement slurry in the soil body to reduce the flow performance, the slurry diffusion range is reduced and in the process of grouting, there is a phenomenon of running slurry in the cement slurry in the shallower grouting position, which results in reductions in the final grouting volume.
2. The diffusion form and range of the CN-1-type geopolymer in the grouting position is different from that of ordinary cement. In this grouting process, the diffusion form of geopolymer slurry is mainly compact diffusion, with a small amount of penetration diffusion, and there is a string slurry phenomenon between each solid body, while the diffusion form of cement slurry is mainly cleavage diffusion with a small amount of penetration diffusion. The different diffusion forms lead to a larger reinforcement area of the geopolymer-grouted pile and a more uniform diffusion of the slurry, thus enhancing the bearing capacity of the model pile.
3. The CN-1-type geopolymer, due to the biased high territory, slag, fly ash and other silica–aluminum-rich source materials, results in the formation of hydration products with a large number of silicon, aluminum, oxygen and alkali metal elements, composing a three-dimensional mesh structure. This structure contributes to the higher strength of the geopolymer solidification body, leading to elevated strength at the interface between the solidification body and the soil. This, in turn, enhances the pile-side friction resistance, ultimately improving the bearing capacity of the model piles.

#### 2.4.2. Test Device and Grouting Process

**Figure 11:**
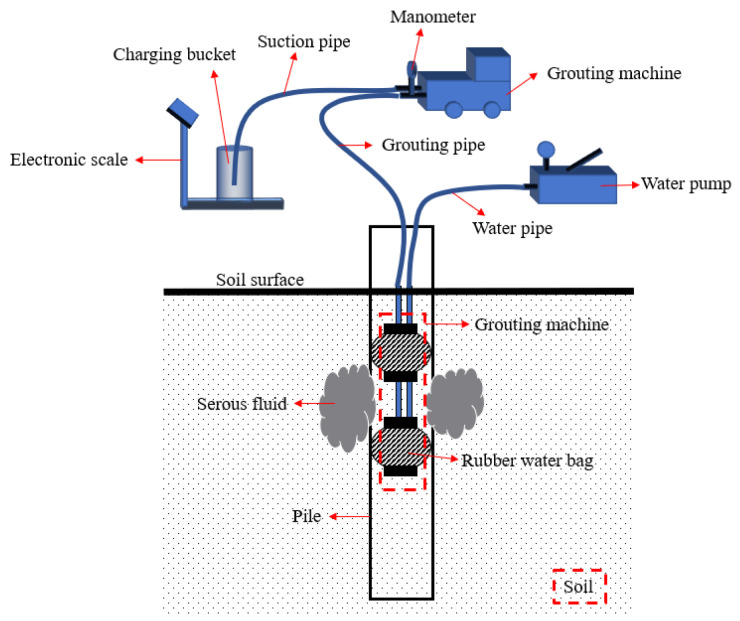
Schematic diagram of distributed grouting system.

**Figure 12:**
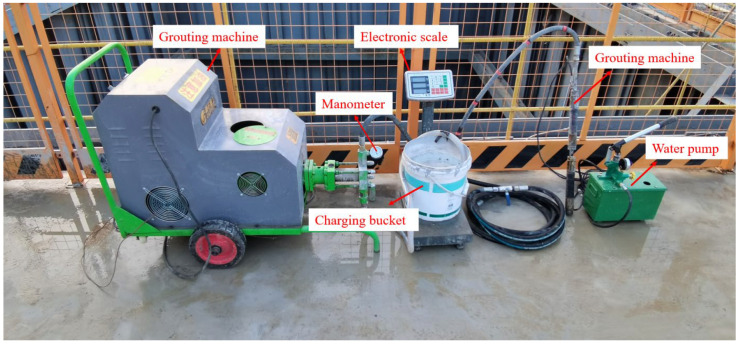
Physical drawing of grouting device.

Super-long piles exhibit typical friction pile characteristics, making pile-side post-grouting a standard method for enhancing their performance [41]. However, due to the traditional ring pipe-type pile-side post-grouting technology’s inherent characteristics, which limits the number of sections grouted to the pile side, the slurry needs to be uniformly distributed along the pile side, resulting in wasted slurry. The number of grouting tubes is large, which is prone to clogging the grouting tubes, and, therefore, it needs to be more applicable to the post-grouting of the pile side of the super-long piles. In this test, a homemade pile-side distributed grouting device is used, as shown in Figure 11 and Figure 12, and it primarily consists of a grouting apparatus, a grouting machine and a water pump. When grouting, the grouting device is first put into the model pile so that its grouting section corresponds to the grouting outlet of the model pile. Then, the rubber airbag on the grouting device is filled with water to make it expand to form a confined space, and then grouting is started. When grouting is completed for the bottom section, the water is drained to make the airbag contract, and then the airbag is upwardly lifted to the next grouting section until grouting is completed.

**Figure 13:**
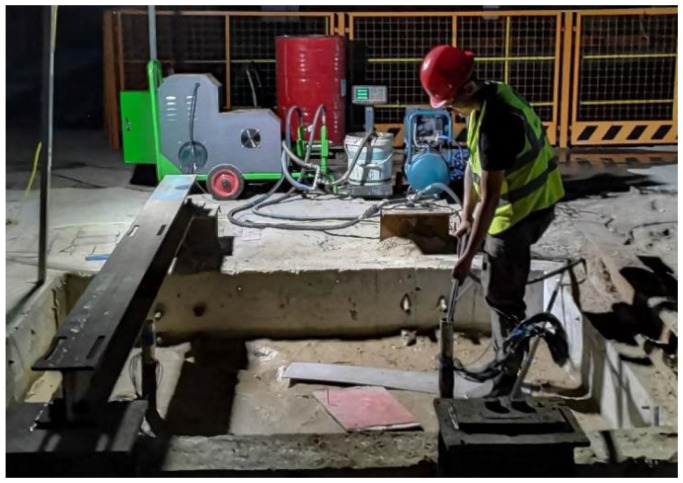
Distributed grouting on pile side.

The grouting process is shown in Figure 13. Grouting commenced three days after the completion of pile formation, and after each grouting section reached the designed grouting volume, the grouting valve was closed. Holding pressure for 2 minutes, the distributed grouting device was manually lifted to the next section until completion. The grouting pressure variation range of P2, P3 and P4 geopolymer-grouted piles was 0.15 MPa~0.25 MPa, and the grouting pressure variation in P1 cement-grouted piles was 0.2 MPa~0.35 MPa. The cement-grouted piles rose faster than the geopolymer-grouted piles in the grouting process, reaching 0.35 MPa, and in the process of grouting, the grouting pressure of cement-grouted piles increased more rapidly than that of geopolymer-grouted piles. In the process of grouting, the bottom grouting of model piles reaches the designed grouting volume, and due to the shallow depth of the top grouting section, there is a phenomenon of bubbling slurry when grouting in the top section of the model piles, which leads to reductions in the grouting volume of the top grouting section.

### 2.5. Vertical Bearing Capacity Test Programs and Devices

#### 2.5.1. Test Programs

For ungrouted piles, loading was carried out three days after the completion of pile formation. For pile-side distributed grouted piles, vertical loading tests were conducted three days after the completion of grouting. The bearing capacity of each pile was predicted before loading and loaded using the slow maintenance loading method according to the Chinese code (JGJ 106-2014) [42]. The predicted ultimate bearing capacity of P0, P1, P2, P3 and P4 piles was 6000 N, 9800 N, 10,500 N, 13,000 N and 14,000 N, respectively. The load grading loading values were 600 N, 980 N, 1050 N, 1300 N and 1400 N, with the first loading level being twice the graded loading value. When the displacement generated under a certain load level is too large or cannot maintain load stability through the supplementary load, it is considered to have reached the ultimate bearing capacity, and the loading is terminated.

#### 2.5.2. Loading Device

**Figure 14:**
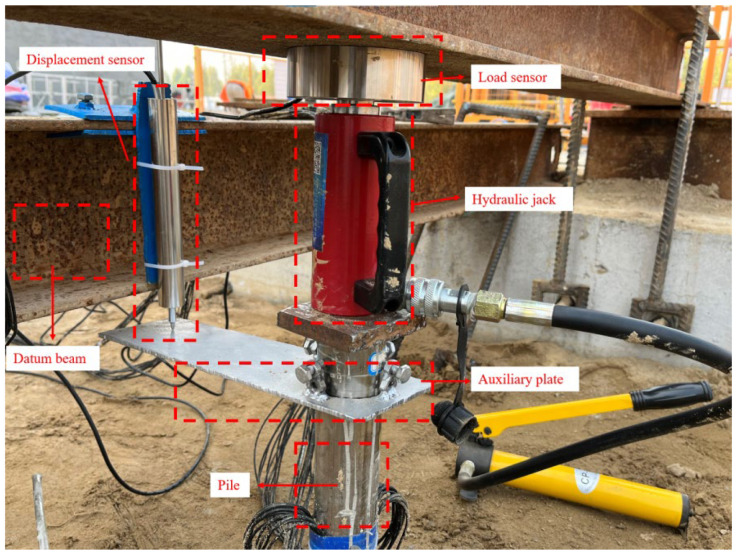
Model pile loading.

**Figure 15:**
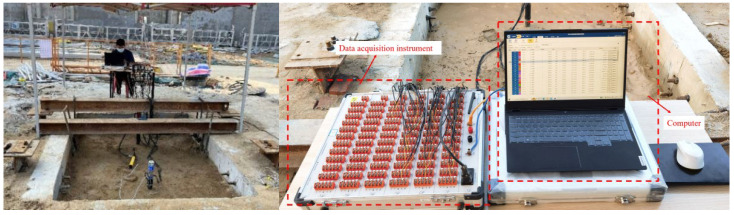
Data acquisition equipment.

This loading employs the self-designed model pile vertical loading system (Figure 14 and Figure 15). The vertical loading system primarily consists of an I-shaped reaction beam, DMWY-100 displacement sensor, displacement meter auxiliary board, load sensor, manual hydraulic jack, data acquisition instrument, computer and other equipment. During loading, the pile top should be polished to ensure the horizontal plane of the pile top.

## 3. Results and Analysis

### 3.1. Pile Top Load–Pile Top Settlement Curve

**Figure 16:**
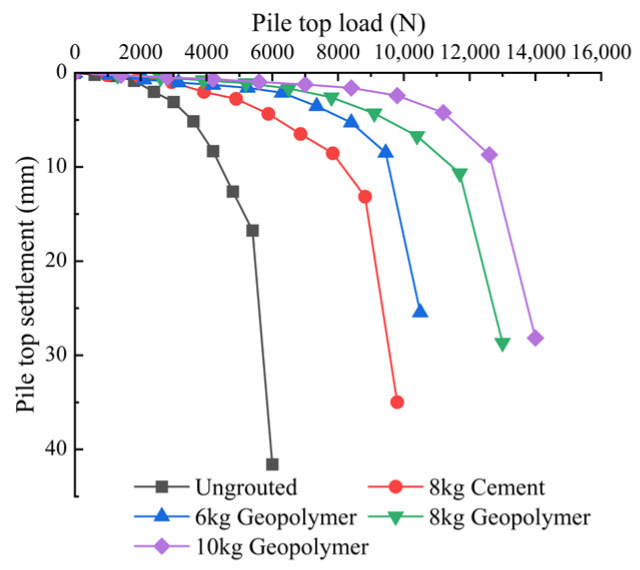
Relationship between pile top load and pile top settlement for each pile.

The pile-top load-top settlement curve is a crucial foundation for characterizing the bearing characteristics of model piles, and the ultimate bearing capacity and damage form of each pile can be determined based on the pile-top load-top settlement curve [43]. The top load-top settlement curve of each pile is shown in Figure 16.

As observed in Figure 16, the load settlement curves for each pile exhibit a “steep decline type,” where the pile’s top sharply descends under the last load. The settlement amount surpasses five-times the upper load, and the pile cannot be stabilized, even after constant supplementary loads, until penetration damage occurs. The ultimate load carrying capacity can be determined for P0, P1, P2, P3 and P4, resulting in values of 5400 N, 8820 N, 9450 N, 11,700 N and 12,600 N, respectively. The ultimate bearing capacity of P0, P1, P2, P3 and P4 can be judged to be 5400 N, 8820 N, 9450 N, 11,700 N and 12,600 N, respectively. The enhancement in the ultimate bearing capacity of P1, P2, P3 and P4 grouted piles compared to ungrouted piles is 163%, 175%, 217% and 233%, respectively. Under the same grouting amount, the ultimate bearing capacity of geopolymer-grouted pile P3 is increased by 133% compared with the cement-grouted pile P1, and the settlement of the pile top is greatly reduced under the same loading level, indicating superior performance of the geopolymer grouting material compared to traditional cement grouting. Its superior suitability for post-grouting on the pile side is evident. Comparing different grouting amounts, the ultimate load capacity of the piles increases with the rising grouting volume, the ultimate load capacity of P3 increased by 124% relative to P2 and that of P4 increased by 108% relative to P3, which indicated that the ability to increase the ultimate load capacity by increasing the amount of grouting is limited. A maximum threshold value exists, beyond which the ultimate load capacity enhancement becomes smaller. In addition, the settlement of ungrouted piles under the first three levels of loading is small, and the settlement from the fourth level of loading is constantly increasing; the settlement of grouted piles is smaller than that of ungrouted piles under the first five levels of loading, and the trend of the curves is slower. Under the maximal load, the settlements of the tops of the piles of P1, P2, P3 and P4 are 84%, 61.2%, 68.9% and 67.7% that of the P0 piles, respectively, and the top settlements of the piles of the grouted piles on the pile side are far smaller than that of the ungrouted piles under the same level of loading. The top settlement of pile-side grouted piles is significantly smaller than that of ungrouted piles, indicating that the distributed grouting on the pile side can not only enhance the ultimate bearing capacity of the piles but also substantially decrease the top settlement of the piles.

### 3.2. Pile Axial Force Distribution Curve with Depth

The axial force transfer characteristics of the pile body are an essential reflection of the bearing characteristics of the single pile. It can not only reflect the exertion of the pile-side friction resistance but also characterize the bearing characteristics of the pile. The axial force of the pile body of the model pile is obtained by the conversion of the data measured by the strain gauge arranged on the side of the pile body. The strain gauge is measured as the strain data, and the average strain value of the section can be obtained by Formula (1):

(1) $$\varepsilon_{i}=\frac{\varepsilon_{i1}+\varepsilon_{i2}}{2}$$

In the formula, $\varepsilon_{i}$ is the average strain of section i and corresponds to the strain values of the strain gauge symmetrically set on the pile body of the section, respectively.

After the average strain value is obtained, the axial force of the section can be obtained from Formula (2):

(2) $$N_{i}=\varepsilon_{i}\times E\times A$$

**Figure 17:**
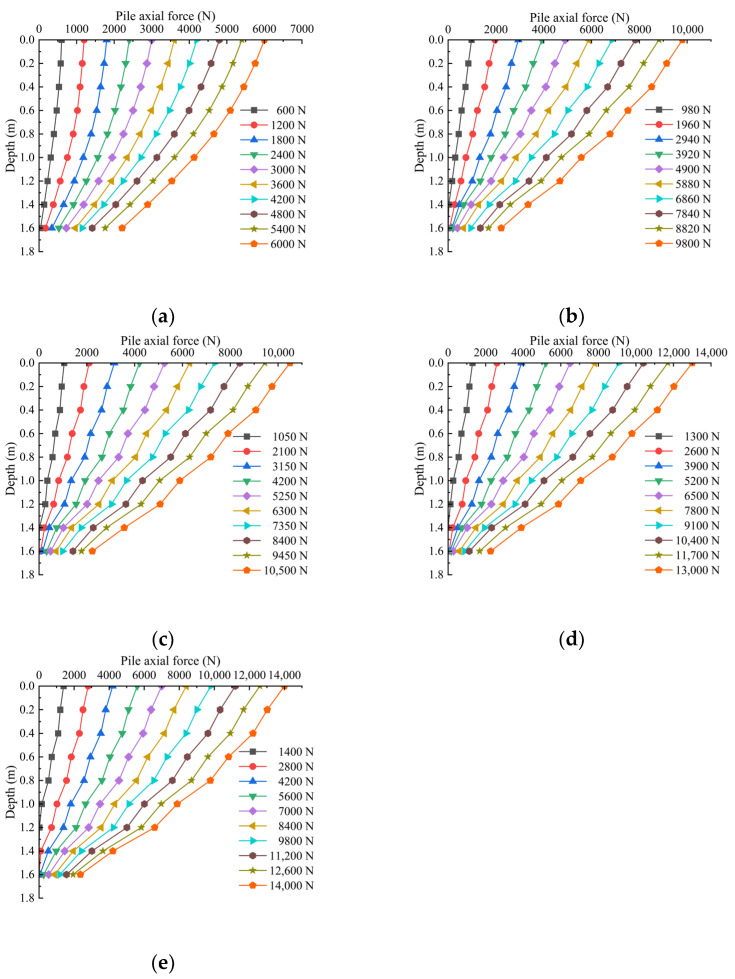
Relationship between axial force distribution of each pile body along depth under various levels of loading. (**a**) Pile axial force–depth distribution curves of ungrouted piles under various levels of loading. (**b**) Pile axial force–depth distribution curve of cement grouted 8 kg pile at pile side under various levels of loading. (**c**) Pile axial force–depth distribution curves of pile-side geopolymer grouted 6 kg piles under various levels of loading. (**d**) Pile axial force–depth distribution curves of 8 kg piles with ground polymer grouting on the pile side under various levels of loading. (**e**) Pile axial force–depth distribution curves of 10 kg piles with ground polymer grouting on the pile side under various levels of loading.

In the formula, $N_{i}$ is the axial force of the pile body in the i section; $\varepsilon_{i}$ is the average strain of section i; E is the elastic modulus of the model pile; A is the cross-sectional area of the model pile. The axial force distribution of each pile with the depth under various loads is shown in Figure 17.

As observed in Figure 17, under all loading levels, the axial force within the pile body of each pile diminishes with increasing depth. This suggests that the upper lateral resistance surpasses the lower play, and with escalating loading, the slope of the axial force curve gradually diminishes. The slope of the axial force curve reflects alterations in lateral friction resistance, and a smaller slope indicates a more pronounced difference in axial force between two sections. Consequently, the greater the lateral friction resistance, the more prominently it is manifested from top to bottom with increasing load. For the ungrouted pile, the axial force curve is relatively smooth, with linear distribution above 0.4 m burial depth, and the slope of the curve decreases in the range of 0.6~1.0 m burial depth, reflecting that the difference in axial force becomes larger in this range, and the lateral friction resistance plays a more considerable role. The axial force of the grouted pile decays faster than that of the ungrouted pile, and in the range of 0.4~0.6 m, 0.8~1.0 m and 1.2~1.4 m, the axial force curve has a sudden change, the slope becomes smaller and with the increase in the grouting depth, the sudden change is more prominent. This is due to the formation of the consolidation body at the pile-side grouting position, and the lower consolidation body is larger than the upper one; the consolidation body has a squeezing effect on the soil around the pile, which makes the effective stress and shear strength of the soil around the pile increase. It strengthens the friction force on the pile side. At the top grouting position, the slope change is not apparent; this is because the top grouting due to the shallow depth led to a more bubbling slurry, did not form a larger solid body and, due to the lower part of the slurry upward return so that the upper part of the model pile to form a layer of the uniform solid body shell, the friction force plays a more uniform role. In addition, the grouted pile has less axial force transfer to the lower part than the ungrouted pile at the early loading stage, which also indicates that the grouting on the pile side will change the stress path of the soil around the pile, making the pile-side resistance to be exerted in advance, which will affect the load transfer characteristics of the pile foundation.

### 3.3. Distribution Curve of Lateral Frictional Resistance of Pile Body along Depth

**Figure 18:**
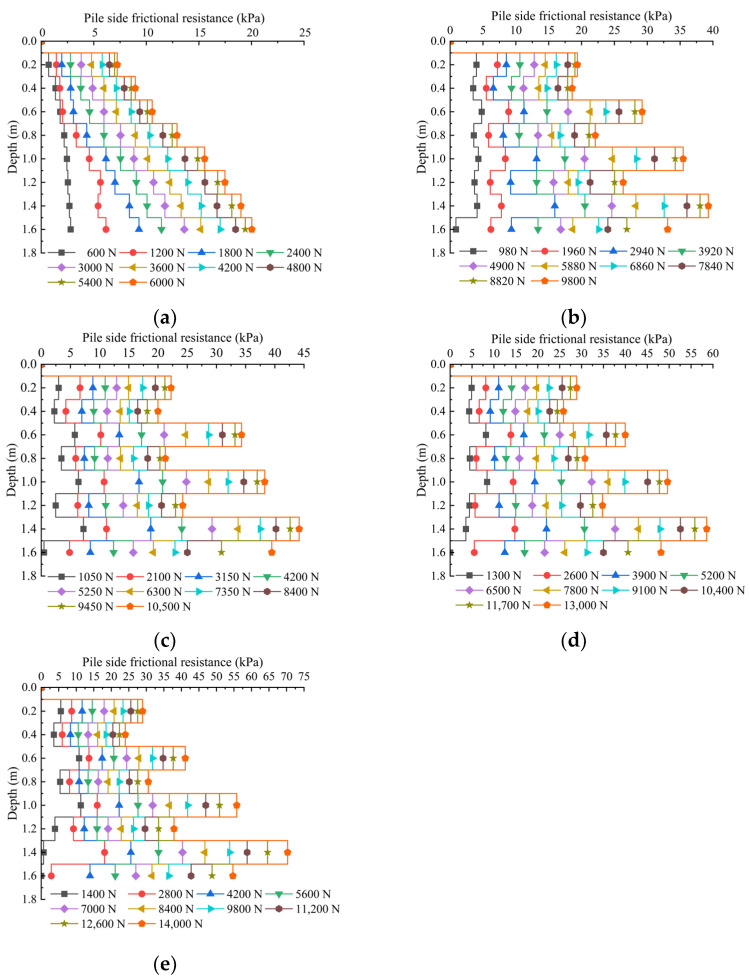
Distribution curve of average side friction resistance along depth for each pile under various levels of loading. (**a**) Average side friction resistance–depth distribution curve of ungrouted piles under various levels of loading. (**b**) Average side friction resistance–depth distribution curve of 8 kg cement grouted piles under various levels of loading. (**c**) Average side friction resistance–depth distribution curve of 6 kg geopolymer-grouted piles under various levels of loading. (**d**) Average side friction resistance–depth distribution curve of 8 kg geopolymer-grouted piles under various levels of loading. (**e**) Average side friction resistance–depth distribution curve of 10 kg geopolymer-grouted piles under various levels of loading.

Based on the obtained axial force data, the average pile lateral friction resistance between sections can be converted according to Equation (3):

(3) $$q_{i}=\frac{N_{i+1}-N_{i}}{\pi DL_{i}}$$

where $q_{i}$ is the average lateral friction resistance of section i; $N_{i+1}$ is the axial force of the pile body in section i+1; $N_{i}$ is the axial force of the pile body in section i; D is the pile diameter; and $L_{i}$ is the length of the pile body in the section. The obtained side friction resistance distribution curve along the depth is shown in Figure 18.

From Figure 18, it can be seen that lateral friction resistance is a process of gradual exertion from top to bottom. In the loading process, the magnitude of the increase in the lateral friction resistance of the pile body is different at different depths. When the load acting on the top of the pile is small, the lateral friction resistance of the top part of the pile body is exerted before that of the bottom. The lower part of the pile body has a minor lateral friction resistance; with the gradual increase in the load on the top of the pile to the ultimate load, the growth rate of the lateral friction resistance of the pile body of the top part of the pile body slows down, and its size tends to stabilize. In contrast, the lateral friction resistance of the pile body of the middle and bottom parts of the pile body continues to increase with the fast growth rate. For ungrouted piles, the lateral friction resistance of the pile body increases with the depth of burial as the load increases. For grouted piles, the distribution law of lateral friction resistance is mostly consistent, and the lateral friction resistance has a sudden change and significant increase at the consolidation body, corresponding to the abrupt change in axial force at the consolidation body. In addition, with the load increase, the lateral friction resistance at the top consolidation body is better than that at the bottom. When the ultimate load is reached, the lateral friction resistance at the bottom consolidation body becomes more prominent than that at the top. This is due to the more significant burial depth of the lower part, and the squeezing effect of the consolidation body on the soil around the pile is more significant than that of the upper part. The effective horizontal stress of the soil around the pile is increased, which results in a significant increase in the lateral friction resistance. Moreover, the sudden change in lateral friction resistance at the top consolidation body is not evident, which is since the upper slurry returns more along the pile body, forming a layer of a homogeneous consolidation body shell on the upper part of the pile body, which makes the upper friction resistance play a more homogeneous role.

**Figure 19:**
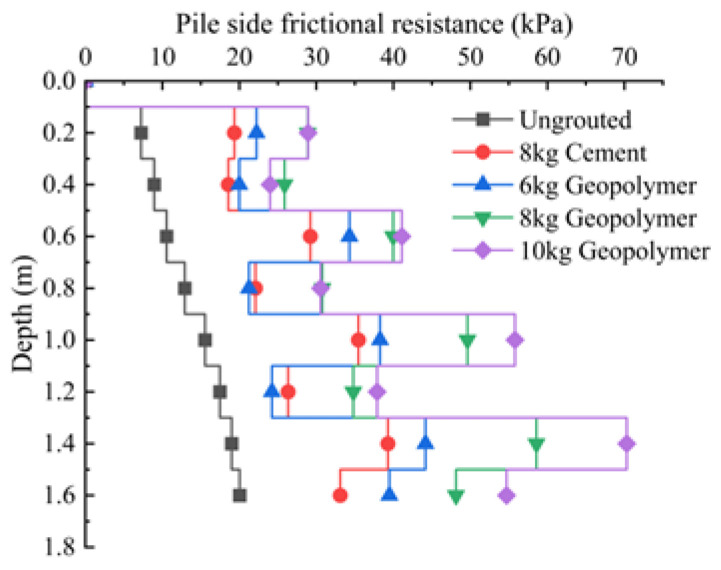
Distribution of side friction resistance of each pile under ultimate load along depth.

The distribution of pile-side friction resistance along the depth for each pile under ultimate load is illustrated in Figure 19. From the figure, it can be seen that under the action of ultimate load, the average values of pile lateral molar resistance of model piles P0, P1, P2, P3 and P4 are 13.96 kPa, 27.93 kPa, 30.49 kPa, 39.58 kPa and 44.59 kPa, and the lateral molar resistance of grouted piles P1, P2, P3 and P4 is dramatically improved compared to that of ungrouted pile P0. The respective enhancement ranges are 200%, 218%, 284% and 319%. This indicates that pile-side grouting effectively improves the physical and mechanical properties of the soil surrounding the pile, enhancing the strength and stiffness of the soil on the pile side. Under the same grouting volume, the ultimate lateral friction resistance of the geopolymer-grouted pile is improved by 142% compared to that of the cement-grouted pile. The settlement of the pile top is smaller under the same load, which indicates that the performance of geopolymer slurry is better than that of traditional cement slurry. The effect of improving the mechanical properties of pile lateral soil is more prominent, and there is a more substantial synergistic effect with the pile lateral soil. When comparing different grouting amounts, the ultimate average lateral resistance increases with the grouting amount, but the rate of increase diminishes, suggesting a limited capacity to enhance ultimate lateral resistance by increasing the grouting amount.

## 4. Diffusion of Slurry around Model Pile

**Figure 20:**
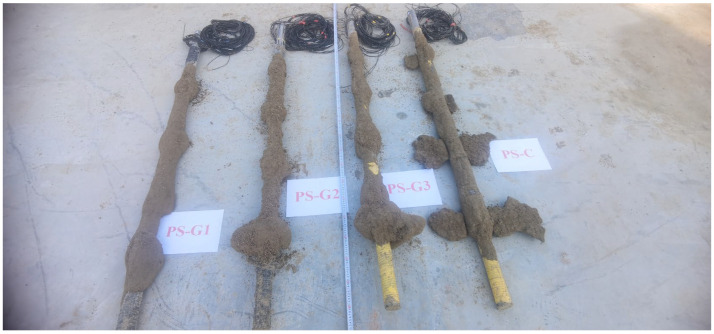
The excavated model pile.

**Figure 21:**
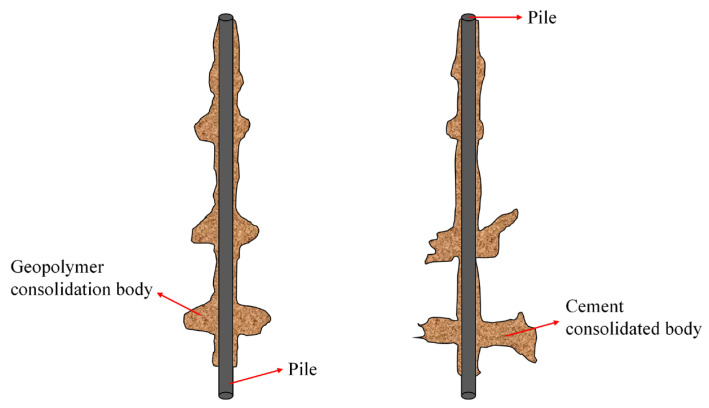
A schematic diagram of geopolymer (**left**) and cement (**right**) slurry diffusion.

After loading, the model pile is excavated, and the slurry distribution on the pile side is observed. The excavation results are depicted in Figure 20 and Figure 21. In the figure, PS-G1, PS-G2, PS-G3 and PS-C represent 6 kg grouting of geopolymer, 8 kg grouting of geopolymer, 10 kg of geopolymer and 8 kg of cement, respectively. The illustrations reveal that in the geopolymer grouting pile, the geopolymer slurry is distributed as an ellipsoid pile at the grouting position. The slurry mainly exhibits compaction and diffusion, accompanied by a small amount of infiltration and diffusion. At the grouting position, the slurry undergoes upward and downward infiltration. The lower consolidation body is the largest, the upper consolidation body is the smallest and the slurry is threaded between the consolidation bodies, resulting in a more uniform distribution of slurry around the pile.

**Figure 22:**
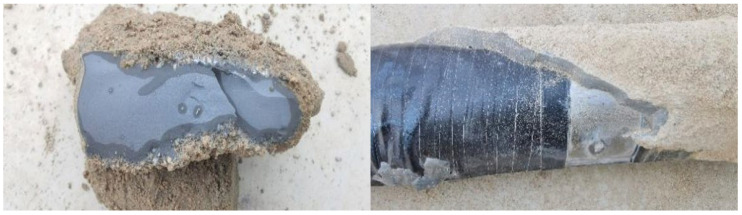
Partial geopolymer consolidation body.

The bonding area between the consolidation body and the pile body is large, augmenting the synergistic interaction between the pile and the surrounding soil. Among them, the consolidation body formed by the PS-G1 pile has a radial diffusion range of 10.4~20.1 cm, and a slurry shell of 0.5~1 cm thickness is formed between the consolidation bodies. The consolidation body formed by the PS-G2 pile has a radial diffusion range of 12~25 cm, and a slurry shell of 0.8~1.3 cm thick is formed between the consolidation bodies. The consolidation body formed by the PS-G3 pile diffuses in a radial range of 12.5~31 cm, and a slurry shell of 0.9~1.5 cm thickness is formed between the solidified bodies. For the cement grouting pile, the slurry is mainly split and diffused in the lower grouting section, forming a flaky splitting consolidation body with a length of about 35~44 cm, a width of about 10.5~13 cm and a thickness of about 2 cm. In the upper grouting section, it is mainly squeezed and diffused, forming an ellipsoidal consolidation body, and the consolidation body occurs between the slurry, forming a layer of slurry shell with a thickness of about 0.8 cm in the pile body. It is noteworthy that following the completion of loading, the model pile undergoes excavation, revealing that the consolidation body of the geopolymer grouting pile is relatively intact and securely bonded to the pile body. In contrast, the fragmented consolidation body of the cement grouting pile results from its limited bonding area with the pile body; the bonding surface of the consolidation body is destroyed during loading. This is also one of the reasons why the ultimate bearing capacity of the geopolymer grouting pile is higher than that of the cement grouting pile. Part of the geopolymer consolidation body is shown in Figure 22.

## 5. Conclusions

This paper presents a model test on the vertical bearing capacity of super-long piles with distributed post-grouting on the pile side. It compares the vertical bearing characteristics of super-long piles in silt fine sand under various grouting materials and amounts. The excavation results of the post-grouting model pile are analyzed to compare the diffusion forms of different grouts in sandy soil, leading to the following conclusions:

1. The ultimate bearing capacity of the ungrouted pile P0 is 5400 N. For the pile-side distributed post-grouting piles (P1, P2, P3 and P4), the ultimate bearing capacities are 8820 N, 9450 N, 11,700 N and 12,600 N, respectively, demonstrating improvements of 163%, 175%, 217% and 233% compared to the ungrouted piles. Under extreme load conditions, the top settlements of P1, P2, P3 and P4 piles were 84%, 61.2%, 68.9% and 67.7% of that observed in P0 piles. Importantly, the top settlements of pile-side grouted piles were significantly smaller than those of ungrouted piles at the same load level, highlighting that distributed post-grouting on the pile side not only enhanced the ultimate load carrying capacity of the piles but also substantially reduced top settlements.
2. Under equivalent grouting amounts, the ultimate bearing capacity of geopolymer-grouted piles is increased by 133% compared to that of normal Portland cement-grouted piles. This observation suggests that, during the same grouting process, geopolymer-grouted piles exhibit superior bearing performance compared to piles grouted with normal Portland cement. This finding validates the applicability of geopolymer grouting materials in post-grouting applications for pile foundations in sandy soil. It serves as a valuable reference for the engineering implementation of post-grouting in pile foundations using geopolymer.
3. When comparing various grouting amounts, an increase in the grouting quantity correlates with an enhanced ultimate bearing capacity of the pile. Specifically, the ultimate bearing capacity of P3 increases by 124% relative to P2, and that of P4 increases by 108% relative to P3. This observation suggests that the capacity to enhance the ultimate bearing capacity by escalating the grouting volume is constrained; there exists a maximum threshold. Beyond this threshold, the incremental growth in the ultimate bearing capacity diminishes in magnitude.
4. Under the influence of the ultimate load, the average values of pile-side friction resistance for P1, P2, P3 and P4 are 27.93 kPa, 30.49 kPa, 39.58 kPa and 44.59 kPa, respectively. The enhancement in pile-side friction resistance, when compared to the ungrouted pile P0, is 200%, 218%, 284% and 319%, indicating that distributed post-grouting on the pile side predominantly enhances the ultimate load carrying capacity by improving the pile-side friction resistance. Moreover, under the same grouting quantity, geopolymer-grouted piles exhibit superior performance in enhancing pile-side friction resistance compared to normal Portland cement-grouted piles. This suggests that distributed post-grouting on the pile side primarily elevates the ultimate bearing capacity by augmenting pile-side friction resistance, and, under equivalent grouting amounts, geopolymer-grouted piles outperform normal Portland cement-grouted piles in enhancing side friction resistance.
5. The geopolymer grouting pile forms an ellipsoidal consolidation body at the pile-side grouting, primarily characterized by compaction diffusion and accompanied by a minor amount of seepage diffusion. The normal Portland cement grouting pile generates a sheet consolidation body at the grouting position in the lower part of the pile body, mainly exhibiting split diffusion. An ellipsoid consolidation body is formed at the grouting position in the upper part of the pile body. The post-grouting piles of both materials exhibit grouting between each grouting section, forming a layer of slurry shell within the pile body. The slurry is evenly distributed along the pile body, thereby increasing the contact area between the pile body and the consolidation body and enhancing pile–soil interaction. The distributed grouting process on the pile side demonstrates excellent applicability to the post-grouting of super-long pile sides.

## 6. Shortages and Prospects

Considering that this model test is conducted on fine sand soil and cannot fully replicate the actual pile-forming method and soil stress state, the results of this test come with certain limitations. However, the primary objective of this test is to compare the bearing capacity of CN-1 geopolymer and ordinary Portland cement model piles under the same test conditions, along with the influence of different grouting amounts on the bearing capacity. As a result, this test can offer valuable insights for the engineering application of the geopolymer. To enhance the validation of practical engineering application, the engineering performance of the CN-1 geopolymer can be further assessed through field tests and numerical analysis.

## Figures and Tables

**Table 1:** Basic physical and mechanical parameters of model soil.

Soil Name	Density (g/cm^3^)	Water Content (%)	Cohesion (kPa)	Internal Friction Angle (°)	Void Ratio	Constrained Modulus (MPa)
Fine silt sand	1.9	24.8	8.8	31.2	0.81	21.2

**Table 2:** Basic parameters of CN-I geopolymer grouting material.

Water-Cement Ratio	Setting Time (Min)		Flow Time (s)	Bleeding Rate (%)	Expansion Ratio (%)	Water Resistance (%)	Constrained Modulus (MPa)		
	Initial	Final					1 d	7 d	28 d
0.6	≥90	≦140	15	≦0.4	0.01	≥95	≥20	≥40	≥50

**Table 3:** Table of grouting schemes for each model pile.

Number	Grouting Methods	Grouting Materials	Grout Amount
P0	Non-grouting	-	-
P1	Pile side	Cement	8 kg
P2	Pile side	Geopolymer	6 kg
P3	Pile side	Geopolymer	8 kg
P4	Pile side	Geopolymer	10 kg

## Data Availability

Dataset available on request from the authors.
